# Good rate of clinical response to cholinesterase inhibitors in mild and moderate Alzheimer's disease after three months of treatment: An open-label study

**DOI:** 10.1590/S1980-57642013DN70200009

**Published:** 2013

**Authors:** Luis Felipe José Ravic de Miranda, Marilourdes do Amaral Barbosa, Patrícia Regina Henrique Peles, Patrícia Hilar Pôças, Pedro Augusto Lopes Tito, Rafael de Oliveira Matoso, Thiago Oliveira Lemos de Lima, Edgar Nunes de Moraes, Paulo Caramelli

**Affiliations:** 1 Post-Graduate Program in Adult Health Applied Sciences, Department of Internal Medicine, Faculty of Medicine, Federal University of Minas Gerais, Belo Horizonte MG, Brazil;; 2 Department of Psychology, FUMEC University, Belo Horizonte MG, Brazil;; 3 Department of Psychology, Salgado de Oliveira University, Belo Horizonte MG, Brazil;; 4 Department of Psychology, Faculty Uninter, Belo Horizonte MG, Brazil;; 5 Faculty of Medicine, Federal University of Minas Gerais, Belo Horizonte MG, Brazil;; 6 Department of Internal Medicine, Faculty of Medicine, Federal University of Minas Gerais, Belo Horizonte MG, Brazil.

**Keywords:** cholinesterase inhibitors, Alzheimer disease, treatment, clinical trial, open-label

## Abstract

Life expectancy in Brazil has increased markedly over the last 30 years. Hence, age-related disorders, such as Alzheimer's disease (AD), warrant special attention due to their high prevalence in the elderly. Pharmacologic treatment of AD is based on cholinesterase inhibitors (ChEI) and memantine, leading to modest clinical benefits both in the short and long-term. However, clinical response is heterogeneous and needs further investigation.

**OBJECTIVE:**

To investigate the rate of response to ChEI in AD after three months of treatment.

**METHODS:**

Patients with mild or moderate dementia due to probable AD or to AD associated with cerebrovascular disease were included in the study. The subjects were assessed at baseline and again after three months of ChEI treatment. Subjects were submitted to the Mini-Mental State Examination (MMSE), Mattis Dementia Rating Scale, Katz Basic Activities of Daily Living, Pfeffer Functional Activities Questionnaire, Neuropsychiatric Inventory and Cornell Scale for Depression in Dementia. Good response was defined by a gain of ≥2 points on the MMSE after three months of treatment in relation to baseline.

**RESULTS:**

Seventy-one patients, 66 (93%) with probable AD and five (7%) with AD associated with cerebrovascular disease, were evaluated. The good response rate at three months was 31.0%, being 37.2% and 21.4% in mild and moderate dementia, respectively. There were no significant differences on most tests, except for improvement in hallucinations, agitation and dysphoria in moderate dementia patients.

**CONCLUSION:**

The rate of good clinical response to ChEI was higher than usually reported. Specific behavioral features significantly improved in the subgroup of moderate dementia.

## Abstract (pt)

A expectativa de vida no Brasil aumentou significativamente nos últimos 30 anos. Desse modo, transtornos relacionados à idade, como a doença de Alzheimer (DA), merecem especial atenção, devido à elevada prevalência. O tratamento farmacológico da DA se baseia nos inibidores da colinesterase (IChE) e na memantina, com melhora modesta em curto e longo prazo. Entretanto, a resposta clínica é heterogênea e necessita maior investigação.

**OBJETIVO:**

Investigar a taxa de resposta aos IChE em pacientes com DA após três meses de tratamento.

**MÉTODOS:**

Pacientes com demência leve ou moderada devida à DA ou DA com doença cerebrovascular foram avaliados antes e após três meses de uso de IChE. Todos foram submetidos ao Mini-Exame do Estado Mental (MEEM), Escala de Demência Mattis, avaliação das atividades básicas de vida diária de Katz, Questionário de Pfeffer, Inventário Neuropsiquiátrico e Escala de Depressão de Cornell. Boa resposta foi definida pelo ganho de ≥2 pontos no MEEM em relação à primeira consulta, após três meses.

**RESULTADOS:**

Setenta e um pacientes, 66 (93%) com DA provável e cinco (7%) com DA associada à doença cerebrovascular, foram avaliados. A taxa de boa resposta clínica em três meses foi de 31.0%, sendo 37,2% e 21,4% na demência leve e moderada, respectivamente. Não houve diferença significativa na maioria dos testes, exceto para melhora de alucinação, agitação e depressão em pacientes com demência moderada.

**CONCLUSÃO:**

A taxa de boa resposta clínica aos IChE foi superior à encontrada na literatura. Observou-se melhora de alguns sintomas comportamentais em pacientes com DA moderada.

## INTRODUCTION

Alzheimer's disease (AD) is the most common neurodegenerative disorder, causing progressive cognitive and functional impairment, and frequently associated with neuropsychiatric symptoms. AD is the main cause of dementia worldwide, according to numerous epidemiological studies.^1,2^

Specific pharmacological treatment of AD is currently based on cholinesterase inhibitors (ChEI) and memantine, which have been shown to be modestly clinically effective, in several randomized controlled trials (RCTs).^3-6^ Response rate to ChEI after at least 12 weeks has been found to be 9% in relation to global improvement and 10% for cognitive symptoms.^7^ Moreover, positive effects of therapeutic doses of ChEI on neuropsychiatric symptoms were observed in 54% of patients,^8^ together with a modest reduction in functional decline compared with placebo.^3^ However, according to Lanctôt et al.,^7^ RCTs usually exclude patients with several comorbidities, and thus naturalistic studies are also important to guide clinical practice.

In this sense, a very large naturalistic study conducted by Raschetti et al.^9^ found that only 17.8% of mild and 15.7% of moderate AD patients treated with ChEI presented good cognitive response after three and nine months in comparison to baseline. However, treatment effects on behavioral symptoms were not evaluated.^9,10^

Most naturalistic studies with ChEI in AD have been conducted in developed countries, with patients having middle to high education and, in general, high socioeconomic level.^9,10^ Since more than 50% of patients with dementia currently live in the developing world and this proportion is set to increase sharply in the near future to reach over 70% by 2025, studies focusing on these specific populations are needed.^9^

In this scenario, the aim of the present naturalistic study was to evaluate the cognitive, functional and neuropsychiatric response rate to ChEI in a group of Brazilian patients with mild and moderate AD after three months of treatment.

## METHODS

This longitudinal study was conducted at the Geriatric Outpatient Clinic of the Hospital das Clínicas at the Federal University of Minas Gerais (UFMG), in Belo Horizonte (MG), Brazil.

The sample comprised patients evaluated from June, 2009 until October, 2011. Patients included fulfilled the National Institute on Aging and the Alzheimer's Association diagnostic criteria of probable AD dementia^11^ or the NINDS-AIREN diagnostic criteria of AD with cerebrovascular disease (AD + CVD).^12^ Patients presented mild or moderate dementia according to the Clinical Dementia Rating (CDR), i.e., CDR 1 or 2, respectively. Patients with different comorbidities, such as high blood pressure, *diabetes mellitus*, osteoporosis, dyslipidemia, among other diseases, were enrolled in the study, provided there were no signs of clinical decompensation. None of the individuals had been treated with ChEI or memantine before study entry. Patients diagnosed with frontotemporal dementia, dementia with Lewy bodies or vascular dementia, as well as those who had already started the treatment, and also patients with CDR 3, were excluded. Illiterate patients were also excluded, due to its major influence on cognitive performance in the selected tests (MMSE and DRS), which could represent a confounding factor for the analysis.

Donepezil, galantamine or rivastigmine were prescribed to the patients according to the clinicians' preferences. All participants were evaluated by one board-certified geriatrician (LFJRM) at baseline and after three months of treatment, as part of an ongoing 12-month responder analysis study of ChEI in AD.

Clinical evaluations were performed at baseline and after three months. The domains examined and the respective evaluation tools were: global cognition (Mini-Mental State Examination^13,14^ - MMSE, and Mat-tis Dementia Rating Scale^15,16^ - DRS), function (Basic activities of daily living - Katz Basic Activities of Daily Living^17^, and instrumental activities of daily living - Pfeffer Functional Activities Questionnaire^18^ - PFAQ), neuropsychiatric symptoms (Neuropsychiatric Inventory^19^ - NPI) and mood (Cornell Scale for Depression in Dementia^20,21^ - CSDD). Regarding NPI score, for the present study the symptoms abnormal eating behaviors and sleep disturbances were not considered due to difficulties in obtaining the caregivers' opinions about how they could quantify the intensity and frequency of these symptoms. The CSDD was applied to patients and caregivers. In the case of caregivers, it refers to depression of the patients.

The rate of good clinical response was determined based on the proportion of patients who gained 2 or more points on the MMSE after three months of treatment in relation to baseline. Neutral response was defined by variations between -1 and +1 on the MMSE score as compared to baseline, while bad response corresponded to a decrease of 2 or more points on the MMSE after three months.

A blood sample was also drawn from the patients on the first consultation for use in DNA extraction and *Apolipoprotein E* (APOE) genotyping.

Data analysis was carried out with the *Statistical Package for the Social Sciences (SPSS) version 17*. Descriptive statistics were used along with the Kolmogorov-Smirnov test to evaluate the distribution of the variables, the Chi-Square test to compare proportions, one-way ANOVA to compare means and the Kruskal-Wallis test to compare medians, adopting a significance level of 5%. The variables with normal distribution are presented as mean and standard deviation values, while the others are shown as median and confidence interval (95%) values.

The study was approved by the Ethics Committee of our institution and all patients and their family caregivers signed a written informed consent form.

## RESULTS

The evaluated sample comprised 71 patients, of which 66 (93%) had AD and five (7%) AD + CVD. In relation to the severity of dementia, 43 patients had mild (CDR 1) and 28 had moderate (CDR 2) dementia. CDR 1 patients (25 women and 18 men) were aged 76.9±6.5 years, with 4.5±4.2 years of education, while CDR 2 patients (20 women and eight men) were aged 77.4±7.6 years and had a mean educational level of 2.6±2.8 years. None of the patients were excluded or died during the three-month study period (Table 1).

**Table 1:** Main characteristics of the population with mild and moderate AD.

		After three monthsof ChEI treatment	
		**CDR 1 (%)**	**CDR 2 (%)**
Age	≤ 69 years	7 (16.3)	5 (17.9)
	70-79 years	24 (55.8)	12 (42.9)
	≥ 80 years	12 (27.9)	11 (39.3)
	Mean age (SD)	76.9 (6.5)	77.4 (7.6)
Gender	Male	18 (41.9)	8 (28.6)
	Female	25 (58.1)	20 (71.4)
Years of schooling	1 to 4 years	30 (69.8)	26 (92.9)
	5 to 8 years	6 (14.0)	0 (0.0)
	9 to 11 years	4 (9.2)	2 (7.1)
	> 11 years	3 (7.0)	0 (0.0)
	Mean (SD)	4.5 (4.2)	2.6 (2.8)
MMSE	≤ 10	-	11 (39.3)
	11 - 20	27 (62.8)	17 (60.7)
	≥ 21	16 (37.2)	-
Comorbidities	(≤ 2)	9 (20.9)	9 (32.1)
	(> 2)	34 (79.1)	19 (67.9)
ChEI	Donepezil	26 (60.5)	19 (67.9)
	Galantamine	8 (18.6)	4 (14.3)
	Rivastigmine	9 (20.9)	5 (17.9)
Antidepressants	Yes	21 (48.8)	16 (57.1)
	No	22 (51.2)	12 (42.9)
Neuroleptics	Yes	6 (16.3)	8 (28.6)
	No	36 (83.7)	19 (71.4)
Benzodiazepines	Yes	3 (7.0)	2 (7.1)
	No	40 (93.0)	25 (92.9)
APOE genotype	ε2ε3	0 (0.0)	2 (7.1)
	ε3ε3	14 (32.6)	13 (46.4)
	ε3ε4	16 (37.2)	8 (28.6)
	ε4ε4	4 (9.3)	0 (0.0)
	Without results	9 (20.9)	5 (17.9)

APOE: Apolipoprotein E gene.

Overall, 31.0% (n=22) of patients were considered good responders according to the adopted criteria. This pattern of response was observed in 37.2% (n=16) of patients with mild dementia and in 21.4% (n=6) of moderate dementia cases (Table 2).

**Table 2:** Antidepressants, neuroleptics and benzodiazepine drugs usage at baseline and after 3 months of ChEI and rate of response (good, neutral and bad response) in mild and moderate dementia.

	ATD bas	ATD 3	NR bas	NR 3	BDZ bas	BDZ 3	Good	Neutral	Bad	Total
Mild dementia	3	17	1	5	1	2	16 (37.2%)	15 (34.9%)	12 (27.9%)	43
Moderate dementia	1	13	1	2	3	3	6 (21.4%)	13 (46.4%)	9 (32.1%)	28
Total	4	30	2	7	4	5	22 (31.0%)	28(39.4%)	21 (29.6%)	71

ChEI: cholinesterase inhibitors; ATD: antidepressants; NR: neuroleptics; BDZ: benzodiazepines; bas: baseline; 3: after three months of treatment.

**Figure 1:**
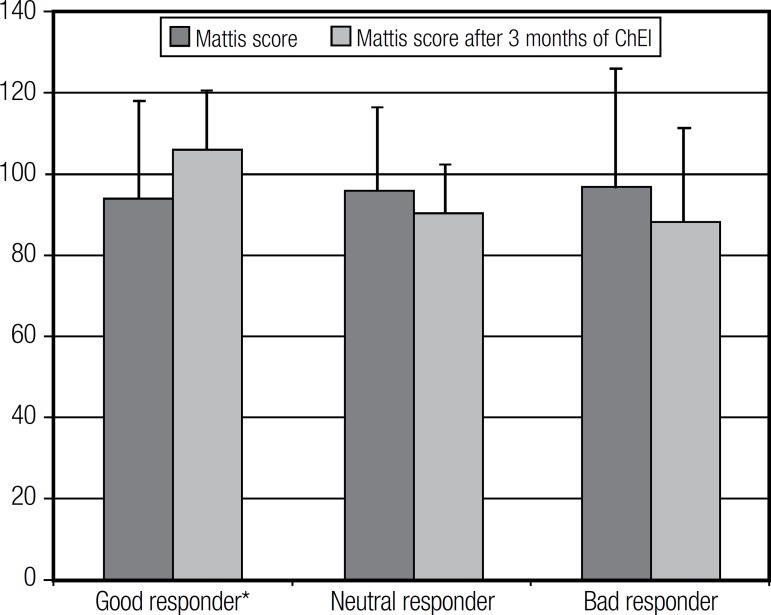
Performance on the Mattis DRS according to response classification before and after 3 months of ChEI.

On average, patients (good, neutral and bad responders) scored 94.9 on the DRS at the first consultation and 95.7 at the end of treatment (Figure 1). The good responders scored 94.1 points on average on the DRS before treatment and 106.1 after three months of ChEI use (p=0.03) (Figure 1).

Some patients classified as mild dementia, whether good, neutral or bad responders, took antidepressants before (T0) and after treatment (T1) (3 at T0 and 17 at T1), neuroleptics (1 at T0 and 5 at T1) or benzodiazepines (1 at T_0_ and 2 at T_1_). Patients with moderate dementia took antidepressants (1 at T0 and 13 at T1), neuroleptics (1 at T0 and 2 at T1) or benzodiazepines (3 at T0 and 3 at T_1_). However, the use of such medications in T_0_ and T1 was not associated with improvement in behavioral symptoms (Table 2).

**Figure 2:**
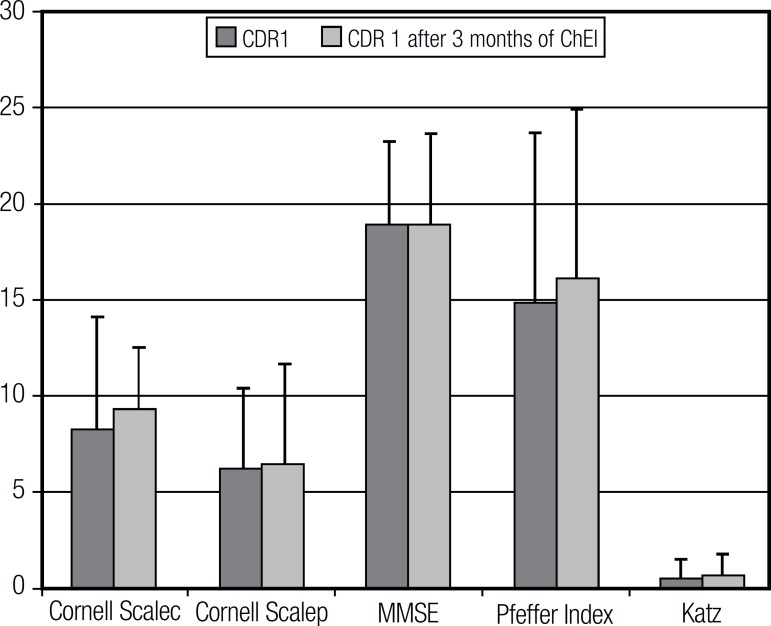
CDR 1 patients' overall performance on cognitive, functional and mood tests before and after 3 months of ChEI treatment.

**Figure 3:**
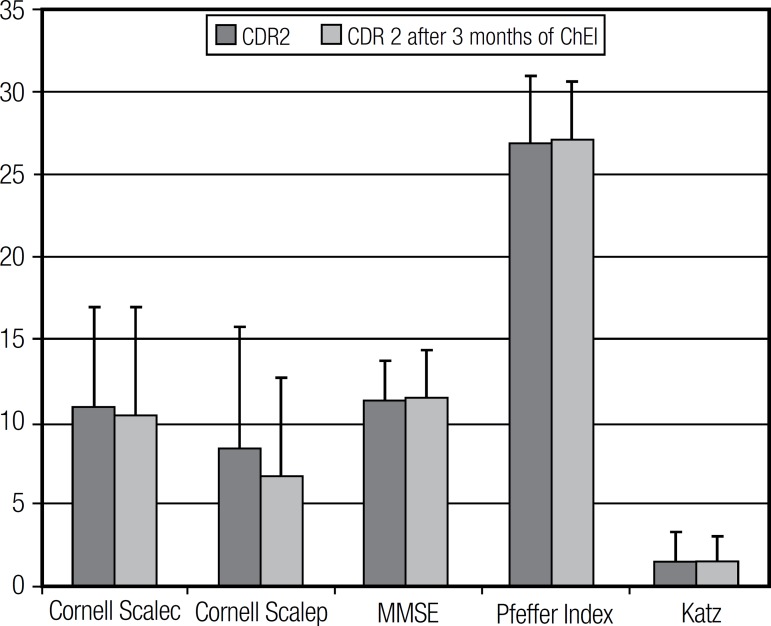
CDR 2 patients' overall performance on cognitive, functional and mood tests before and after 3 months of ChEI treatment.

Among the CDR 1 subgroup, no significant difference was detected after three months of treatment with ChEI in comparison to baseline on any of the tests (Figure 2; Table 3). Similarly, in the CDR 2 subgroup, no statistically significant difference was observed after three months of ChEI on any of the tests applied, except for the NPI, which showed that moderate dementia patients had a significant reduction in hallucinations, agitation and dysphoria (Figure 3; Tables 4 and 5).

**Table 3:** Neuropsychiatric Inventory (NPI) scores before and after 3 months of treatment in mild AD patients (CDR 1).

NPI domains	Baseline	After 3 monthsof ChEI	p-value
Delusion	30.2%	20.9%	p>0.05
Hallucinations	14.0%	16.3%	p>0.05
Agitation	39.5%	37.2%	p>0.05
Dysphoria	60.5%	55.8%	p>0.05
Anxiety	53.5%	51.2%	p>0.05
Euphoria	9.3%	9.3%	p>0.05
Apathy	53.5%	46.5%	p>0.05
Disinhibition	23.3%	20.9%	p>0.05
Irritability	48.8%	53.5%.	p>0.05
Aberrant motor activity	23.3%	20.9%	p>0.05

ChEI: cholinesterase inhibitors.

**Table 4:** Neuropsychiatric Inventory (NPI) scores before and after 3 months of treatment in moderate AD patients (CDR 2).

NPI domains	Baseline	After 3 months of ChEI	p-value
Delusion	32.0%	36.0%	p>0.05
Hallucinations	46.0%	25.0%	p=0.031
Agitation	75.0%	54.0%	p=0.031
Dysphoria	79.0%	64.0%	p=0.043
Anxiety	57.0%	46.0%	p>0.05
Euphoria	7.0%	4.0%	p>0.05
Apathy	71.0%	71.0%	p>0.05
Disinhibition	36.0%	29.0%	p>0.05
Irritability	50.0%	57.0%	p>0.05
Aberrant motor activity	50.0%	50.0%	p>0.05

ChEI: cholinesterase inhibitors.

**Table 5:** Comparison of functional, mood (patients and caregivers) and MMSE scores between baseline and after 3 months of ChEI treatment in mild (CDR 1) and moderate (CDR 2) AD patients.

	General								CDR 1								CDR 2						
	**Baseline**				**Three months**				**Baseline**				**Three months**				**Baseline**				**Three months**		
	**N**	**M**	**SD**		**N**	**M**	**SD**		**N**	**M**	**SD**		**N**	**M**	**SD**		**N**	**M**	**SD**		**N**	**M**	**SD**
Katz	71	0.9	1.4		71	1.1	1.3		43	0.5	1.0		43	0.6	1.1		28	1.6	1.7		28	1.7	1.4
PFAQ	70	19.5	9.4		71	20.5	8.9		43	14.9	8.8		43	16.2	8.8		27	26.9	4.1		28	27.1	3.5
CSDD Patient	66	7.0	5.5		68	6.6	5.5		43	6.3	4.1		42	6.5	5.3		23	8.5	7.3		26	6.7	6.0
CSDD Caregiver	70	9.3	6.0		69	8.7	5.7		43	8.2	5.9		42	7.5	4.9		27	11.0	6.0		27	10.5	6.5
MMSE	71	15.9	5.2		71	16.0	5.3		43	19.0	4.3		43	18.9	4.5		28	11.3	2.4		28	11.5	2.8

Katz: Basic activities of daily living; PFAQ: Pfeffer Functional Activities Questionnaire; CSDD: Cornell Scale for Depression in Dementia; MMSE: Mini-Mental State Examination; N: number of patients; M: mean value; SD: standard deviation.

## DISCUSSION

In this open-label naturalistic study evaluating the effects of ChEI treatment over three months in patients with mild to moderate AD, a good rate of clinical (cognitive) response was observed. Overall, almost one third of the cases presented cognitive benefits as measured by the MMSE. Moreover, significant positive effects on behavior were observed in the subgroup of cases with moderate dementia. Some caution, however, must be taken in the interpretation of these findings, due to methodological limitations of the study (see below).

Regarding symptomatic effects on the different domains affected, no improvements were seen in the whole population in relation to cognition (DRS and MMSE), functional performance (PFAQ), neuropsychiatric features (NPI) or mood (Cornell). In the analysis conducted according to dementia severity, no significant benefits emerged in CDR 1 patients, while in CDR 2 cases significant improvement was seen on specific neuropsychiatric features, namely, hallucinations, agitation and depression.

The rate of good responders found in this study is higher than rates previously reported by most investigators, ranging from 9% to 15.7%.^7,9^ Raschetti et al.,^9^ after three months of treatment with ChEI, found that the pattern of clinical response by the end of this period was a good predictor of the response found at nine months.

Good responders performed better on the DRS. In this work, the DRS was used as one of the cognitive measures of efficacy. In a previous meta-analysis of 16 RCTs with ChEI,^7^ the authors found a lower cognitive (9%) and global (10%) response compared to the present study. However, these investigators included in the analysis RCTs lasting from 12 to 52 weeks, thus precluding a direct comparison with the present study data.

According to many investigations,^7,8,22-24^ the use of ChEI improves cognition, behavioral symptoms and functional performance, thus diminishing caregiver stress. Even treated patients who deteriorate, lose fewer points on the MMSE than those who do not receive appropriate pharmacological treatment.^25^

The majority of studies published thus far span six to nine months of treatment. Although several of these trials included open-label extensions, it is difficult to ascertain how long treatment with ChEI sustains the cognitive state of patients.^9,26,27^

In the current study, no changes in cognitive performance were observed by the end of the three-month period, as measured by the MMSE - from 15.9±5.2to 16.0±5.3(CDR 1 and CDR 2), from 19.0±4.3to 18.9±4.5in CDR 1 and from 11.3±2.4to 11.5±2.8in CDR 2 patients (Table 5). Similarly, mood symptoms (CDSD), instrumental (PFAQ) and basic (Katz scale) activities of daily living did not change significantly with treatment.

In the beginning of ChEI use, physicians and researchers had great enthusiasm with these medications, because of the well-established clinical benefits, which brought hope to patients as well as their families and caregivers. This treatment represents a landmark in the clinical management of patients with AD. However, although significant in many RCTs, the effects proved to be modest in clinical practice and with great heterogeneity in individual response.

Holmes et al.^8^ and Vogel et al.^28^ stressed the positive effects of ChEI in improving neuropsychiatric symptoms in AD, as measured by the NPI, although neither of them compared the frequency of NPI symptoms between CDR 1 and CDR2.

As previously mentioned, RCTs in dementia and AD usually last less than one year, typically spanning six months.^7^ Hence, information on maintenance of long-term benefits in the domains of cognition, mood, behavior and activities of daily living is relatively scarce. Moreover, Lanctôt et al.^7^ stated that meta-analyses may suffer from publication bias, since studies with a positive result tend to be more published than negative studies, resulting in an overestimate of treatment efficacy.

In the present study, mild AD patients presented dysphoria (61%), apathy (55%), and irritability (50%) as the most frequent neuropsychiatric symptoms. A reduction in their frequency was observed, albeit without reaching statistical significance. Among patients with moderate dementia, dysphoria (79%), agitation (75%), apathy (50%), pacing (50%), anxiety (57%), irritability (50%), and hallucinations (46%) were the most common behavioral symptoms. Dysphoria, agitation, and hallucinations significantly improved after three months of treatment with ChEI. Dysphoria improvement was not related to the use of antidepressants. We found scant studies evaluating behavioral effects of ChEI treatment in mild and moderate patients separately. However, a recent study by Kavanagh et al.^29^ concluded that galantamine improves NPI scores in moderate AD, but not in mild AD, only after five to six months of treatment. These same good results were not seen after three months of treatment. Improvement in behavioral symptoms were observed in other studies on patients treated with ChEI.^29-31^

A higher rate of good response was observed in this study than in previous reports, both in mild (37.2%) and moderate (21.4%) dementia. These results do not allow any solid conclusions to be drawn because the sample is small and the follow-up period short. Further analysis, based on the results from a larger sample after 12 months of treatment, should provide more robust information.

The limitations of this work were basically the small sample (71 patients) and the short follow-up period (three months). An increase in sample size and a longer follow-up shall provide more reliable data.

In conclusion, the study found a modest, albeit significant, improvement with ChEI treatment in cognition and behavior in a subset of mild and moderate AD patients. We believe that doctors should be aware of the true range of effectiveness of pharmacological treatment in dementia, in order to better orient patients and their families during the course of the illness.
